# Proteome changes in the small intestinal mucosa of broilers (*Gallus gallus*) induced by high concentrations of atmospheric ammonia

**DOI:** 10.1186/s12953-015-0067-4

**Published:** 2015-02-21

**Authors:** Jize Zhang, Cong Li, Xiangfang Tang, Qingping Lu, Renna Sa, Hongfu Zhang

**Affiliations:** State Key Laboratory of Animal Nutrition, Institute of Animal Sciences, Chinese Academy of Agricultural Sciences, Beijing, 100193 People’s Republic of China

**Keywords:** Proteome, Ammonia, Small intestinal mucosa, Broilers

## Abstract

**Background:**

Ammonia is a well-known toxicant both existing in atmospheric and aquatic system. So far, most studies of ammonia toxicity focused on mammals or aquatic animals. With the development of poultry industry, ammonia as a main source of contaminant in the air is causing more and more problems on broiler production, especially lower growth rate. The molecular mechanisms that underlie the negative effects of ammonia on the growth and intestine of broilers are yet unclear. We investigated the growth, gut morphology, and mucosal proteome of Arbor Acres broilers (*Gallus gallus*) exposed to high concentrations of atmospheric ammonia by performing a proteomics approach integrated with traditional methods.

**Results:**

Exposure to ammonia interfered with the development of immune organ and gut villi. Meanwhile, it greatly reduced daily weight gain and feed intake, and enhanced feed conversion ratio. A total of 43 intestinal mucosal proteins were found to be differentially abundant. Up-regulated proteins are related to oxidative phosphorylation and apoptosis. Down-regulated proteins are related to cell structure and growth, transcriptional and translational regulation, immune response, oxidative stress and nutrient metabolism. These results indicated that exposure to ammonia triggered oxidative stress, and interfered with nutrient absorption and immune function in the small intestinal mucosa of broilers.

**Conclusions:**

These findings have important implications for understanding the toxic mechanisms of ammonia on intestine of broilers, which provides new information that can be used for intervention using nutritional strategies in the future.

**Electronic supplementary material:**

The online version of this article (doi:10.1186/s12953-015-0067-4) contains supplementary material, which is available to authorized users.

## Background

Ammonia is a colorless and highly water-soluble gas, which is a well-known toxicant both in aquatic and atmospheric system. In animal houses, ammonia may be formed mainly from animal manure by hydrolysis, mineralization, and volatilization [1]. Animal produced ammonia accounts for almost 50% of the total annual anthropogenic emission of ammonia_,_ in which poultry operations produced the highest ammonia emission as compared with other animal groups [2,3]. The limiting level of ammonia for poultry is under 25 μL/L. But in practice, birds are often exposed to higher concentrations of ammonia (50–200 μL/L) in some poorly ventilated facilities. High level of atmospheric ammonia induces several problems in broiler production, such as decreased growth rate, body weight, and increased feed conversion [4,5]. Longtime exposure can create many health issues in broilers and severely interfere with broiler welfare [6,7].

In previous research, degenerative vacuole and necrosis of renal tubulae were observed in livers and kidneys of ammonia-exposed broilers, respectively [8]. Apoptosis of epithelium cells of tracheal mucosa has been demonstrated in ammonia-exposed broilers in our study (unpublished data). The neurotoxicity of ammonia induces an increase in expression of tumor necrosis factor α (TNF-α) and interleukin 1 β (IL-1β), which can be associated with the production of reactive oxygen species (ROS), nitric oxide (NO) involved with protein kinase A (PKA), extracellular signal regulated kinase (ERK) pathway and nuclear factor-κB (NF-κB) activation in astrocytes in rats [9].

Negative effects of ammonia on gastrointestinal (GI) tract were also reported in previous studies that may be related to nutrient metabolism and energy production. In rat colonocytes, it showed that ammonia suppressed short-chain fatty acid (SCFA) oxidation [10]. Inhibition of oxygen consumption induced lower energetic efficiency and decreased cellular energy production were also observed in the similar animal model due to elevated concentration of ammonia by the ingestion of high protein diet [11]. Tsujii et al. [12] reported that ammonia impaired mitochondrial and cellular respiration, and energy metabolism of gastric mucosa, which triggered a decrease of mucosal cell viability leading to mucosal damage subsequently. Moreover, Igarashi et al. [13] demonstrated that ammonia accelerated cytokine-induced apoptosis in human gastric epithelial cell lines.

Gastrointestinal (GI) tract is regarded as the essential sensory organ for nutrition absorption, immune response, and pathogen prevention [14]. Previous research have demonstrated that changes of animal growth performance are closely related to alterations of protein expression in the small intestinal mucosa [15-17]. There are numerous enzymes in the small intestinal mucosa involved in different physiological functions, such as protein metabolism, lipid metabolism, carbohydrates metabolism, energy production, mucosal integrity and so on [18-21]. However, it is almost impossible to detect a huge number of proteins in the GI mucosa at the same time using traditional methods, for example western blots, immunohistochemical staining or ELISAs. Currently, most studies relevant to toxic mechanisms of ammonia are on mammals and aquatic animals. Little is known about the alteration of proteins in the small intestinal mucosa of broilers that have been exposed to high concentrations of atmospheric ammonia.

Based on previous research, we hypothesized that high concentrations of atmospheric ammonia exposure can confer negative effects on growth via changes of proteins involved in different physiological processes in the small intestinal mucosa of broilers, which requires further study to elucidate. Therefore, in this study, we utilized a label-based iTRAQ procedure (isobaric tags for relative and absolute quantitation), followed by LC-MS/MS to quantitate altered proteins that are induced differentially in the small intestinal mucosa of broilers exposed to high concentrations of atmospheric ammonia.

## Materials and methods

### Animals and exposure conditions

A total of 60 1-day-old Arbor Acers (AA) male broilers were obtained from a commercial hatchery in Beijing (Beijing Arbor Acers Broiler Co., Beijing, China). All birds were housed in individual wire-bottom cages in an environmentally controlled room under standard brooding practices, and given *ad libitum* access to water and a maize-soybean basal diet during the first 21 days. Then, broilers were transferred to environmentally controlled exposure chambers. The diet during the experiment was formulated to achieve the National Research Council (NRC, 1994) recommended requirements for all nutrients containing ME, 12.76 MJ kg^−1^; and crude protein 19.94% (Additional file 1: Table S1). The concentrated ammonia was delivered in a whole-body animal exposure chamber [7] from days 22 to 42. Each exposure chamber was a 4500 × 3000 × 2500 mm (length × width × height) sealed unit, sectioned for housing 30 birds per chamber. Temperature and airflow were controlled during the exposures to ensure adequate ventilation, minimize buildup of animal-generated contaminants (dander, H_2_S, CO_2_) and to avoid thermal stress [22].

The setting of the concentration of ammonia in the present study was according to previous studies that the growth performance of broilers was severely interfered with ammonia level over 70 μL/L [4,7,8]. Treatment (TRET) group of broilers were exposed to 75 ± 3 μL/L ammonia during the experimental period. Control (CTRL) broilers were raised in a separated chamber without ammonia for the same period, and the concentration of ambient ammonia was kept at 3 ± 3 μL/L. The concentration of ammonia in both chambers was monitored with a LumaSense Photoacoustic Field Gas-Monitor Innova-1412 (Santa Clara, CA, USA) during the entire experimental period. Body weight (BW) and feed consumption were recorded weekly for feed-conversion ratio evaluation. This study was carried out in strict accordance with the Regulations for the Administration of Affairs Concerning Experimental Animals of the State Council of the People’s Republic of China. The protocol was approved by the Committee on Experimental Animal Management of Chinese Academy of Agricultural Sciences.

### Sample collection

At day 42, all birds were weighed after a 12 h-fasting (12 h food withdrawal) period. The growth parameters (n = 30) including body weight gain, feed intake and feed-conversion ratio were determined. Twelve birds (6 per each group) were randomly selected for blood and small intestine sample collection. Each blood sample was obtained from a wing vein using a sterilized syringe within 30 s. Blood was incubated in a water bath for 1 h at 37°C then centrifuged at 400 × g for 10 min at 4°C, and the sera obtained were stored at −80°C for further analysis [23]. After blood sampling, the chickens were sacrificed by cervical dislocation and then exsanguinated. Immediately after death, the intestinal mucosa was scraped from the intestinum tenue with the back of a surgery knife as described by Luo et al. [24], frozen in liquid nitrogen, and stored at −80°C for further proteome and qPCR analyses. Samples of about 1 cm of medial duodenum (apex of the duodenum), medial jejunum (midway between the point of entry of the bile duct and Meckel’s diverticulum) and medial ileum (midway between Meckel’s diverticulum to the ileocecal junction) were taken and fixed in buffered 4% formal-saline solution before processing for embedding in paraffin. To calculate the indices of immune organs, another twelve birds (6 per each group) were killed as described above, and the bursa of Fabricius, spleen, thymus and intestine of were excised and weighted, respectively.

### Biochemical and histological analyses

For biochemical analysis, the activities of creatine kinase (CK) and total superoxide dismutase (T-SOD) in the serum were measured using a corresponding diagnostic kit (Nanjing Jiancheng Bioengineering Institute, Nanjing, China) according to the instructions of the manufacturer. Histological examination was carried out according to the method described by [25]. Briefly, villus height was determined from the tip of the villus to the villus crypt junction and crypt depth was defined as the depth of invaginations between adjacent villi.

### Small intestinal mucosa preparation and protein extraction

Sample pooling is a commonly used strategy to reduce the influence of individual variation on candidate target selection in proteomic studies [24,26,27]. To avoid erroneous conclusions due to individual variations, the same amount of the intestinal mucosa (weight: weight as 1: 1 ratio) from two chickens in the same group was pooled as a biological replicate, and three biological replicates were acquired for each group.

Each pooled small intestinal mucosal sample (~0.5 g) was ground in a Dounce glass grinder using liquid nitrogen. Ground samples were precipitated with 10% trichloroacetic acid (TCA) (w/v), 90% ice-cold acetone at −20°C for 2 h. The samples were then centrifuged at 20,000 × g for 30 min at 4°C. The supernatants were decanted and the pellets washed with ice-cold acetone. The pellets were lysed in lysis buffer consisting of 8 M urea, 30 mM 4-(2-hydroxyethyl)-1-piperazineethanesulfonic acid (HEPES), 1 mM phenylmethanesulfonyl fluoride (PMSF), 2 mM ethylene diamine tetraacetic acid (EDTA), and 10 mM dithiothreitol (DTT). The crude tissue extracts were centrifuged for 30 min at 20,000 × g to remove the undissolved pellets. The tissue lysates were reduced for 1 h at 36°C in water bath by addition of 1 M DTT to a final concentration of 10 mM DTT and then alkylated for 1 h by addition of 1 M iodoacetamide (IAM) to a final concentration of 55 mM in the dark. After reduction and alkylation, proteins were precipitated by adding 4 volumes of ice-cold acetone. The pellets were then washed three times with ice-cold pure acetone and resuspended in buffer consisting of 50% tetraethyl ammonium bromide (TEAB) and 0.1% sodium dodecyl sulfonate (SDS). The samples were then centrifuged for 30 min at 20,000 × g and the undissolved pellets were removed and protein quantitation performed using a Bio-Rad Bradford Protein Assay Kit (Hercules, CA, USA).

### Trypsin digestion, iTRAQ labeling and strong cation exchange chromatography

Modified sequence grade trypsin (Promega Corporation, Madison, WI) was added to each sample at a 1:30 ratio (3.3 μg trypsin : 100 μg target) and digested overnight at 37°C.

Each isobaric tag was solubilized in 70 μL isopropanol. Tags (113, 114, 115, 116, 117 and 121) were added to respective pooled samples (3 pooled replicates in each group) individually and incubated at room temperature for 2 h. Additional isopropanol was added to samples to ensure an organic composition > 60% prior to incubation.

The strong cation exchange fractionation protocol followed a previous report [28] with slight modification. Briefly, the samples were loaded onto a strong cation exchange column (Phenomenex Luna SCX 100A) equilibrated with buffer A (10 mM KH_2_PO_4_ in 25% acetonitrile, pH 3.0) using an Agilent 1100 (Santa Clara, CA) system. The peptides were separated using a linear gradient of buffer B (10 mM KH_2_PO_4_ and 2 M KCl in 25% acetonitrile, pH 3.0) increasing to 5% after 36 min, 50% after 66 min and 100% after 71 min, at a flow rate of 1 ml/min. Elution was monitored by setting the absorbance at 214 nm. The eluted peptides were pooled into 10 fractions, desalted with a Strata X C18 column (Phenomenex) and vacuum-dried.

### Mass spectrometry

Each fraction was resuspended in buffer A (2% acetonitrile, 0.1% formic acid) and centrifuged at 20,000 × g for 10 min. In each fraction, the final concentration of peptides was approximately 0.25 μg/μl. Using an autosampler, 20 μl of supernatant was loaded onto a 2 cm C18 trap column (inner diameter 200 μm) on an UltiMate® 3000 Nano LC system (Bannockburn, IL). Peptides were eluted onto a resolving 100 mm × 75 μM analytical C18 column containing 5-μm particles that was assembled in-house. Samples were loaded at 15 μl/min for 4 min and eluted with a 45-min gradient at 400 nl/min from 5 to 60% buffer B (98% acetonitrile, 0.1% formic acid), separated with a 3 min linear gradient to 80% B, maintained at 80% B for 7 min, returned to 5% B over 3 min, and finally combined with a Q-Exactive mass spectrometer (Thermo Scientific, MA, USA). The mass spectrometer was operated in data dependent acquisition mode, with MS performed in the Q-Exactive at a resolution of 70,000 full width at half maximum (FWHM). MS/MS was performed in high-energy collision dissociation (HCD) operating mode and product ions were detected in the Q-Exactive at 17,500 FWHM resolution. Data were acquired using a data-dependent data acquisition mode in which, for each cycle, the ten most abundant multiply charged peptides (2^+^ to 4^+^) with an m/z between 350 and 2000 were selected for MS/MS with a 15-s dynamic exclusion setting.

### Data processing and analyses

For iTRAQ protein identification, the raw mass data were processed with Proteome Discover 1.3 (Thermo Fisher Scientific) and searched with in-house MASCOT software (Matrix Science, London, U.K.; version 2.3.0) against the database Uniprot_Gallus gallus_9031 (Apr 11th, 2014) the following parameters: enzyme: trypsin; fixed modification: carbamidomethyl (C); variable modifications: oxidation (M), gln-pyro-glu (N-term Q), iTRAQ 8-plex (N-term, K, Y); peptide tolerance:15 ppm; MS/MS tolerance: 20 mmu; maximum missed cleavages: 1. All identified peptides had an ion score above the Mascot peptide identity threshold, and a protein was considered identified if at least one such unique peptide match was apparent for the protein. For iTRAQ quantitation, MASCOT software was also used. Protein quantitative values were derived only from uniquely assigned peptides. Intra-sample channels were normalized based on the median ratio for each channel across all proteins. Ratios for each iTRAQ label were obtained using a pooled sample in the control group (sample tagged with 113) as the denominator. Inter-sample, protein reference, and spectrum normalizations were performed. Differential expression in the TRET samples was then presented as a log_2-fold_ change relative to the CTRL. Thus, the fold change for each individual reporter ion is based on referencing a reporter channel which is then log transformed to base 2. Proteins were deemed to be differentially expressed using Student’s *t*-test corrected for multiple testing using the Benjamini and Hochberg correction [29]. Proteins with a 1.2-fold change or greater were considered to be differentially expressed.

### Bioinformatics analysis of proteins differential abundance

Gene Ontology (GO) distribution for all of the proteins that were significantly altered in the small intestinal samples of ammonia exposed chickens were classified using Blast2GO software (http://www.blast2go.com/) and WEGO (http://wego.genomics.org.cn) that were provided by the Institute for Genomic Research [30,31].

### Validation of proteins of differential abundance

Real-time quantitative PCR (qPCR) was used to verify seven intestinal mucosal proteins of differential abundance at the mRNA level.

Total RNA from intestinal mucosal samples was isolated using a Qiagen RNeasy Plus Mini Kit (Valencia, CA). The quality of the RNA was evaluated by electrophoresis on an agarose gel, and the quantity of the RNA was measured with a spectrophotometer (Nanodrop 2000, Thermo Scientific, Waltham, MA).

Reverse transcription was performed immediately following total RNA isolation using PrimeScript™ Reverse Transcriptase, D2680A (Takara, Dalian, China). RT-qPCR was performed using an Applied Biosystems 7500 Fast Real-Time PCR System (Foster City, CA). RT-qPCRs were performed at 95°C for 30 s, followed by 40 cycles of 95°C for 10 s and 60°C for 30 s. SYBR green fluorescence was detected at the end of each cycle to monitor the amount of PCR product. A standard curve was constructed using a 10-fold dilution series, and its slope was used to calculate the efficiency of the qPCR primers. Primer sequences are listed in Additional file 2: Table S2.

The relative amount of a target gene mRNA was calculated as previously described [23]. The expression level of a target gene mRNA was normalized to the mRNA level of β-actin. The ΔΔ*C*_T_ was calibrated against an average from the control group. The linear amount of the target gene expression to the calibrator was calculated by 2^−ΔΔ*C*T^. Therefore, all gene expression results are reported as the fold difference between treated and control groups. The specificity of the real-time PCR product was verified using a melting curve and DNA sequencing.

### Statistical analysis

Data on growth parameters, immune organ indices, serum parameters, gut morphological structure and gene expressions were analyzed by one-way ANOVA (SAS Version 9.2, SAS institute Inc., Cary, NC). A group difference was assumed to be statistically significant when *P* < 0.05. All results were expressed as means ± S.D.

## Results

### Body weight gain, feed intake, feed-conversion ratio and immune organ indices

The body weight gain and feed intake are key parameters to assess the growth of animal. In this study, all birds (CTRL and TRET) started at the same age (d 22). During the entire experimental period (20 days), TRET birds had 15.4% less (*P* < 0.05) body weight gain and 9.6% less (*P* < 0.05) feed intake. On the contrary, feed-conversion ratio (FCR) in TRET group was greatly increased (*P* < 0.05) compared with CTRL group (Table 1). Of four tested immune organs, indices of spleen and intestine of chickens in TRET group were lighter than those of CTRL group (*P* < 0.05). Thus, exposure to high concentrations of atmospheric ammonia interfered with immune organ development of AA broilers and resulted in a reduction of feed conversion efficiency [24].

**Table 1:** **Effect of atmospheric ammonia on the body weight gain, feed intake, feed-conversion ratio and immune organ indices of broilers**

	**Groups**	
	**Control**	**Treatment**
Body weight gain (g/day)	91 ± 3.6^a^	77 ± 2.5^b^
Feed intake (g/day)	150 ± 1.9^a^	135 ± 2.8^b^
Feed-conversion ratio^c^	1.64 ± 0.09^b^	1.75 ± 0.05^a^
Index of bursal (%)^d^	0.73 ± 0.06	0.61 ± 0.05
Index of spleen (%)^d^	1.07 ± 0.04^a^	0.75 ± 0.02^b^
Index of thymus (%)^d^	2.35 ± 0.50	2.19 ± 0.44
Index of intestine (%)^d^	3.70 ± 0.15^a^	2.67 ± 0.11^b^

^a, b^Values within a row not sharing a common superscript letter indicate significant difference at *P* < 0.05. Numbers are means ± S.D. (n = 30 for growth parameters; n = 6 for indices of immune organs).

^c^Feed-conversion ratio = the ratio of feed intake to body weight gain.

^d^The ratio of organ weight to body weight.

### Serum parameters and gut morphological structure

**Figure 1:**
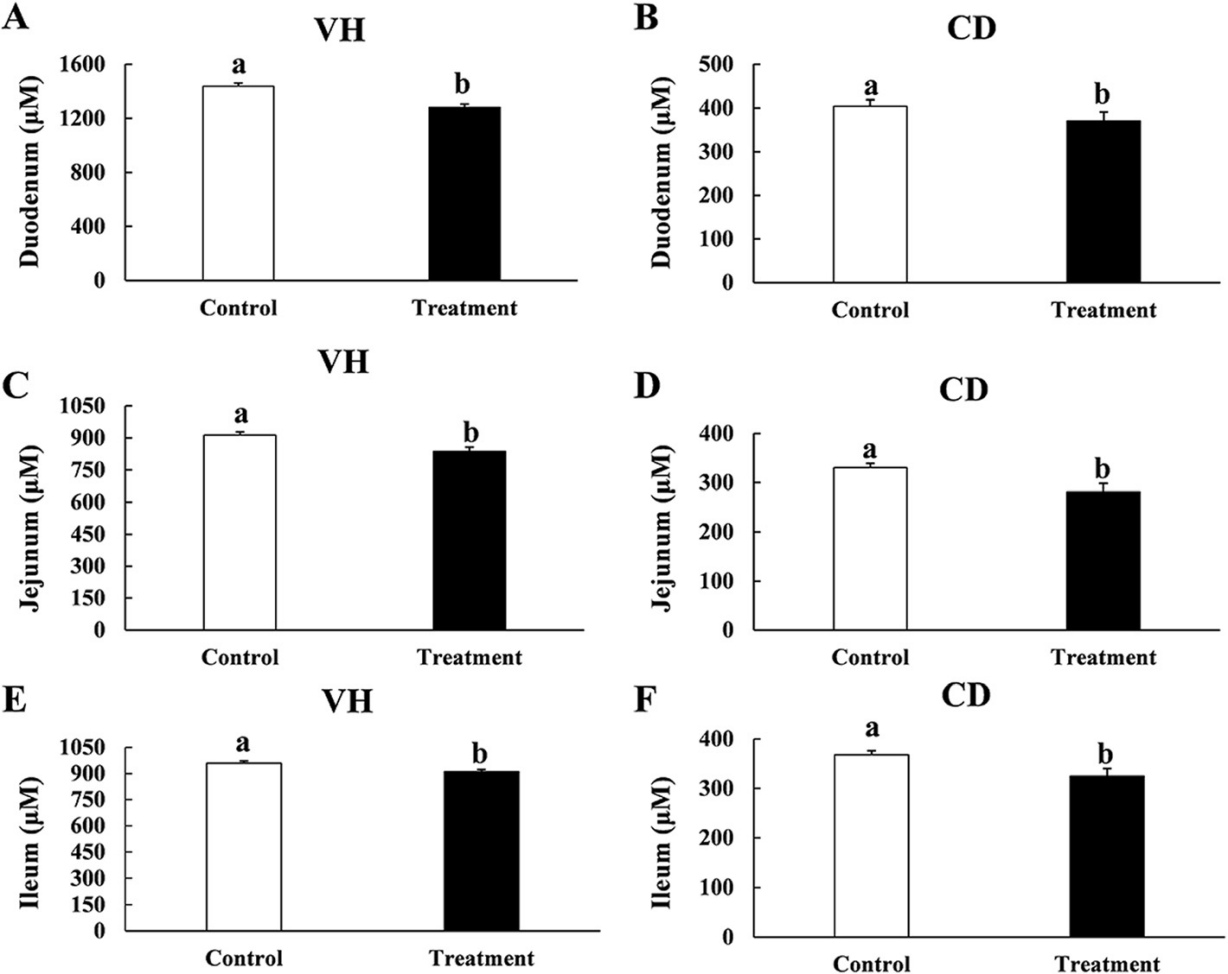
**Effects of atmospheric ammonia on villus height (VH) and crypt depth (CD) of duodenum (A and B), jejunum (C and D) and ileum (E and F) in control and treatment groups.** Vertical lines represent ± S.D, and different letters denote significant difference at *P* < 0.05 (n = 6).

Activity of serum CK is an important indicator under stress in the body [25,32]. T-SOD represents the oxidation resistance in the animal [33]. In TRET broilers, activity of serum CK was significantly elevated (*P* < 0.05) compared with the control group indicating extensive organ injury (Table 2). Antioxidase T-SOD was decreased significantly compared with the control group (*P* < 0.05), illustrating lower oxidation resistance (Table 2). As shown in Figure 1A, B, C, D, E and F the VH and CD in all small intestinal segments of birds in CTRL group were significantly higher than those in TRET group, which implicates the absorptive area of small intestine was greatly reduced in broilers under high level of ambient ammonia.

**Table 2:** **Effect of atmospheric ammonia on the serum biochemical parameters of broilers**

	**Groups**	
	**Control**	**Treatment**
CK (U/L)^c^	6224.50 ± 172.26^b^	7173.63 ± 309.05^a^
T-SOD (U/mL)^d^	77.81 ± 6.55^a^	61.12 ± 2.11^b^

^a, b^Values within a column not sharing a common superscript letter indicate significant difference at *P* < 0.05. Numbers are means ± S.D. (n = 6).

^c^CK = creatine kinase.

^d^T-SOD = total superoxide dismutase.

### Identification and comparison of proteins of differential abundance

Using iTRAQ analysis, a total of 2726 proteins were identified within the FDR of 1% (Additional file 3: Table S3). Following statistical analysis, 70 proteins were found to be differentially expressed in the small intestinal mucosa between CTRL and TRET broilers, with 26 being up-regulated and 44 down-regulated (Additional file 4: Table S4).

**Figure 2:**
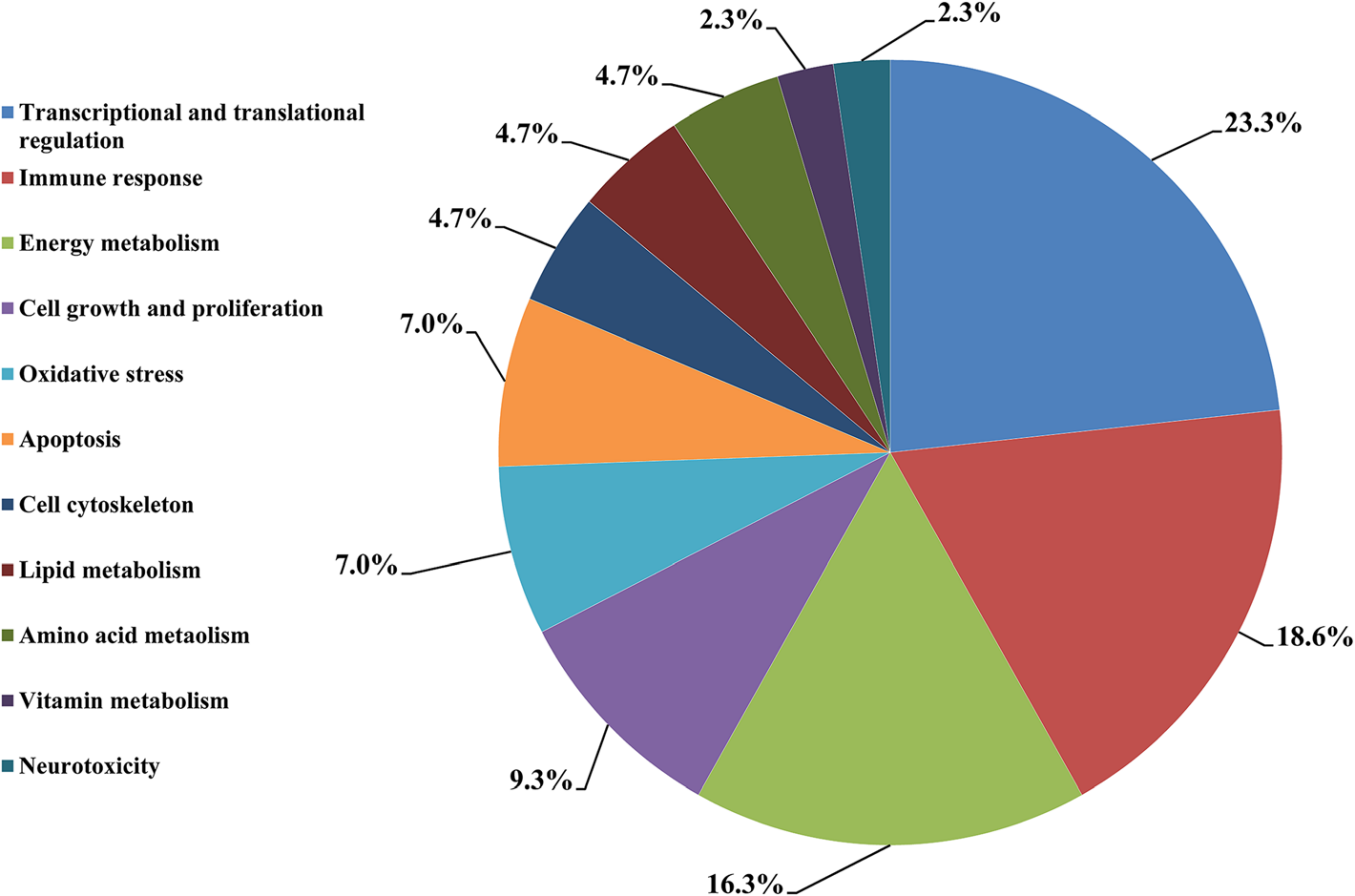
**Functional classification of the proteins of differential abundance identified from the small intestinal mucosa of 42-day-old broilers.**

A total of 43 proteins of differential abundance were grouped into eleven classes based on putative functions: transcriptional and translational regulation (23.3%), immune response (18.6%), energy metabolism (16.3%), cell growth and proliferation (9.3%), oxidative stress (7.0%), apoptosis (7.0%), cell cytoskeleton (4.7%), lipid metabolism (4.7%), amino acid metabolism (4.7%), vitamin metabolism (2.3%) and neurotoxicity (2.3%) (Figure 2). Those related to transcriptional and translational regulation, immune response and energy metabolism were predominant and accounted for approximately 60% of the differentially-expressed proteins. A comparison of proteins of differential abundance with functional grouping between the groups indicated that fewer protein species were up-regulated in ammonia-exposed chickens (11 versus 32, respectively) (Table 3). These 11 up-regulated protein species were distributed in five categories: four in energy metabolism, three in apoptosis, two in transcriptional and translational regulation, one in oxidative, and one in neurotoxicity. The 32 down-regulated protein species were distributed in nine categories: eight in transcriptional and translational regulation, eight in immune response, four in cell growth and proliferation, three in energy metabolism, two in oxidative stress, two in lipid metabolism, two in amino acid metabolism, two in cell cytoskeleton and one in vitamin metabolism.

**Table 3:** **List of differentially expressed proteins in small intestinal mucosal samples from treatment group and control group**

**Accession** ^**a**^	**Description** ^**b**^	**Gene symbol**	**Theoretical MW** ^**c**^ **/pI** ^**d**^	**Score** ^**e**^	**Pep. no.** ^**f**^	**Log**_**2**_ **fold change** ^**g**^	***P-*** **value**	**Biological process GO term**
Energy metabolism								
F1NTZ0	Uncharacterized protein (Fragment) OS = Gallus gallus GN = ADH6 PE = 3 SV = 2 - [F1NTZ0_CHICK]	ADH6	39.5/7.49	565.75	14	−0.60	0.0162	Oxidoreductase activity
R4GI36	Phosphoenolpyruvate carboxykinase, cytosolic [GTP] OS = Gallus gallus GN = PCK1 PE = 3 SV = 1 - [R4GI36_CHICK]	PCK1	61.3/7.75	64.06	2	−0.35	0.0038	Gluconeogenesis
P07322	Beta-enolase OS = Gallus gallus GN = ENO3 PE = 1 SV = 3 - [ENOB_CHICK]	ENO3	47.2/7.61	259.39	5	−0.29	0.0497	Gluconeogenesis
E1C4U7	Uncharacterized protein OS = Gallus gallus GN = NDUFB3 PE = 4 SV = 1 - [E1C4U7_CHICK]	NDUFB3	11.1/9.82	35.42	1	0.39	0.0179	Electron transport chain
E1BQJ6	Uncharacterized protein OS = Gallus gallus PE = 3 SV = 2 - [E1BQJ6_CHICK]		257.7/5.17	23.38	1	0.87	0.0490	ATP binding
Q9YHT1	Succinate dehydrogenase [ubiquinone] flavoprotein subunit, mitochondrial OS = Gallus gallus GN = SDHA PE = 1 SV = 2 - [SDHA_CHICK]	SDHA	72.9/7.08	773.5	18	1.02	0.0211	Succinate dehydrogenase (ubiquinone) activity
F1NZI4	Uncharacterized protein OS = Gallus gallus GN = ATHL1 PE = 4 SV = 2 - [F1NZI4_CHICK]	ATHL1	77/5.73	30.07	1	1.15	0.0239	Catalytic activity
Lipid metabolism								
Q2MJT5	Fatty acid translocase OS = Gallus gallus PE = 2 SV = 1 - [Q2MJT5_CHICK]	CD36	52.6/8.37	110.81	4	−0.45	0.0471	Lipid uptake
E1BS15	Uncharacterized protein OS = Gallus gallus GN = ACSF2 PE = 4 SV = 2 - [E1BS15_CHICK]	ACSF2	68.7/8.7	441.08	15	−0.33	0.0192	Fatty acids catalytic activity
Amino acid metabolism								
P00368	Glutamate dehydrogenase 1, mitochondrial OS = Gallus gallus GN = GLUD1 PE = 1 SV = 1 - [DHE3_CHICK]	GLUD1	55.7/8.28	641.29	19	−0.50	0.0155	Glutamate dehydrogenase [NAD(P)+] activity
F1P3F9	Glutamate dehydrogenase OS = Gallus gallus GN = GLUD1 PE = 3 SV = 2 - [F1P3F9_CHICK]	GLUD1	47.6/8.18	695.44	19	−0.39	0.0288	Glutamate dehydrogenase [NAD(P)+] activity
Vitamin metabolism								
F1P4K4	Uncharacterized protein OS = Gallus gallus GN = ALDH8A1 PE = 3 SV = 2 - [F1P4K4_CHICK]	ALDH8A1	53.2/7.83	31.26	1	−0.54	0.0080	9-cis-retinoic acid biosynthetic process
Cell cytoskeleton								
Q90WF1	Filamin OS = Gallus gallus PE = 2 SV = 1 - [Q90WF1_CHICK]	FLNA	272.8/6.35	103.29	2	−0.72	0.0018	Actin-binding
D2Z1L9	LIM and SH3 protein 1 OS = Gallus gallus GN = LASP1 PE = 2 SV = 1 - [D2Z1L9_CHICK]	LASP1	29.6/7.4	417.26	11	−0.43	0.0284	Zinc ion binding
Cell growth and proliferation								
R4GF89	Uncharacterized protein OS = Gallus gallus GN = SFN PE = 3 SV = 1 - [R4GF89_CHICK]	SFN	27.7/5.01	114.99	3	−0.54	0.0239	Positive regulation of cell growth
E1B	Uncharacterized protein OS = Gallus	RP	14.9/1	286.	8	−0.46	0.0	Structural
Y89	gallus GN = RPL23 PE = 3 SV = 2 - [E1BY89_CHICK]	L23	0.51	2			019	constituent of ribosome
F1NQS9	Uncharacterized protein OS = Gallus gallus GN = ZNF598 PE = 4 SV = 2 - [F1NQS9_CHICK]	ZNF598	99.6/8.57	28.5	1	−0.42	0.0041	Zinc ion binding
E1BTA6	Uncharacterized protein OS = Gallus gallus GN = SEPT12 PE = 3 SV = 2 - [E1BTA6_CHICK]	SEPT12	47.1/8.68	52.61	2	−0.33	0.0202	GTP binding
Transcriptional and translational regulation								
F1P0C0	Uncharacterized protein OS = Gallus gallus GN = HMGN1 PE = 4 SV = 1 - [F1P0C0_CHICK]	HMGN1	11.1/9.26	68.36	1	−0.55	0.0223	Chromatin binding
E1C9F0	Uncharacterized protein OS = Gallus gallus GN = DYNC2H1 PE = 4 SV = 2 - [E1C9F0_CHICK]	DYNC2H1	491.7/6.43	22.2	1	−0.50	0.0062	Protein processing
E1BT82	Uncharacterized protein OS = Gallus gallus GN = EIF2S2 PE = 2 SV = 1 - [E1BT82_CHICK]	EIF2S2	37.9/6.13	71.23	4	−0.44	0.0040	Translation initiation factor activity
Q5ZJ39	Density-regulated protein OS = Gallus gallus GN = DENR PE = 2 SV = 1 - [DENR_CHICK]	DENR	22.1/5.21	60.49	1	−0.41	0.0080	Translation initiation factor activity
F1NS60	Uncharacterized protein (Fragment) OS = Gallus gallus GN = MMS19 PE = 4 SV = 2 - [F1NS60_CHICK]	MMS19	111.8/5.99	55.46	1	−0.38	0.0038	Transcription coactivator activity
E1C4N0	Uncharacterized protein OS = Gallus gallus GN = RPS10 PE = 4 SV = 2 - [E1C4N0_CHICK]	RPS10	18.9/10.15	183.89	6	−0.36	0.0068	Structural constituent of ribosome
R4GL23	Uncharacterized protein OS = Gallus gallus GN = CHTOP PE = 4 SV = 1 - [R4GL23_CHICK]	CHTOP	26.3/12.23	83.09	2	−0.30	0.0036	Transcription export complex
F1NLT8	Uncharacterized protein (Fragment) OS = Gallus gallus GN = ARHGDIB PE = 4 SV = 1 - [F1NLT8_CHICK]	ARHGDIB	23.2/5.2	235.16	7	−0.29	0.0435	Rho GDP-dissociation inhibitor activity
F1NA55	Eukaryotic translation initiation factor 2A OS = Gallus gallus GN = EIF2A PE = 2 SV = 2 - [F1NA55_CHICK]	EIF2A	62.7/8.79	62.63	1	0.27	0.0449	Translation initiation factor activity
Q800W4	TIA-1 OS = Gallus gallus GN = TIA1 PE = 2 SV = 1 - [Q800W4_CHICK]	TIA1	41.3/7.72	62.06	1	0.29	0.0074	Nucleotide binding
Immune response								
P40618	High mobility group protein B3 OS = Gallus gallus GN = HMGB3 PE = 2 SV = 3 - [HMGB3_CHICK]	HMGB3	23/8.12	126.17	4	−0.59	0.0009	DNA binding
Q9YH06	High mobility group protein B1 OS = Gallus gallus GN = HMGB1 PE = 1 SV = 1 - [HMGB1_CHICK]	HMGB1	24.9/5.74	165.42	6	−0.44	0.0084	DNA binding
E1C9I0	Unconventional myosin-Ig OS = Gallus gallus GN = MYO1G PE = 4 SV = 2 - [E1C9I0_CHICK]	MYO1G	115/8.78	203.91	7	−0.47	0.0410	Nucleotide-binding(motor activity)
E1BTE2	Uncharacterized protein OS = Gallus gallus GN = SERPINB5 PE = 3 SV = 2 - [E1BTE2_CHICK]	SERPINB5	42.6/5.96	218.9	7	−0.40	0.0017	Serine-type endopeptidase inhibitor activity
Q90643	Interferon regulatory factor 3 OS = Gallus gallus GN = IRF3 PE = 2 SV = 1 - [IRF3_CHICK]	IRF3	54.4/5.21	45.52	2	−0.33	0.0460	Activation of innate immune response
E1BWS0	Uncharacterized protein OS = Gallus gallus GN = GIT2 PE = 4 SV = 2 - [E1BWS0_CHICK]	GIT2	84.5/6.98	29.12	1	−0.32	0.0461	ARF GTPase activator activity
E1BUY6	Uncharacterized protein OS = Gallus gallus GN = HMHA1 PE = 4 SV = 2 - [E1BUY6_CHICK]	HMHA1	103.3/7.97	29.79	1	−0.31	0.0080	GTPase activator activity
E1BVP2	Uncharacterized protein OS = Gallus gallus GN = PLD1 PE = 4 SV = 2 - [E1BVP2_CHICK]	PLD1	118.9/8.98	50.12	2	−0.27	0.0397	Defense response to Gram-positive bacterium
Apoptosis								
I3VQH4	Interleukin enhancer binding factor 3-like protein OS = Gallus gallus GN = ILF3 PE = 2 SV = 1 - [I3VQH4_CHICK]	ILF3	95/8.81	68.88	3	0.30	0.0025	Participate in the apoptosis
R4GLP0	Uncharacterized protein OS = Gallus gallus GN = COX7C PE = 4 SV = 1 - [R4GLP0_CHICK]	COX7C	7.2/10.96	27.81	1	0.55	0.0380	Cytochrome-c oxidase activity
F1NIC5	Uncharacterized protein (Fragment) OS = Gallus gallus GN = TIMM8A PE = 4 SV = 2 - [F1NIC5_CHICK]	TIMM8A	12.2/7.78	135.92	4	0.57	0.0346	Protein transport (metal ion binding)
Oxidative stress								
H9L201	Uncharacterized protein OS = Gallus gallus GN = PNKD PE = 3 SV = 2 - [H9L201_CHICK]	PNKD	48/9.35	31.96	1	−0.46	0.0095	Glutathione biosynthetic process
F1NQC3	Glutamine synthetase OS = Gallus gallus GN = GLUL PE = 3 SV = 2 - [F1NQC3_CHICK]	GLUL	46.5/7.72	479.53	13	−0.40	0.0483	Glutamate-ammonia ligase activity
P08267	Ferritin heavy chain OS = Gallus gallus GN = FTH PE = 2 SV = 2 - [FRIH_CHICK]	FTH	21.1/6.21	311.58	8	0.34	0.0248	Ferroxidase activity
Neurotoxicity								
F1P4B2	Protein piccolo (Fragment) OS = Gallus gallus GN = PCLO PE = 4 SV = 2 - [F1P4B2_CHICK]	LOC768552	560.2/6.77	33.63	1	0.62	0.0247	cAMP-mediated signaling

^a^Uniprot_Gallus gallus_9031 database accession number.

^b^The name of the protein exclusive of the identifier that appears in the database.

^c^Theoretical molecular mass (kDa).

^d^Theoretical pI.

^e^The sum of the scores of the individual peptides.

^f^The number of distinct peptide sequences in the protein group.

^g^Differential protein expression in the treatment group was presented as a log_2_ fold change relative to the control group.

### GO annotations of proteins of differential abundance

**Figure 3:**
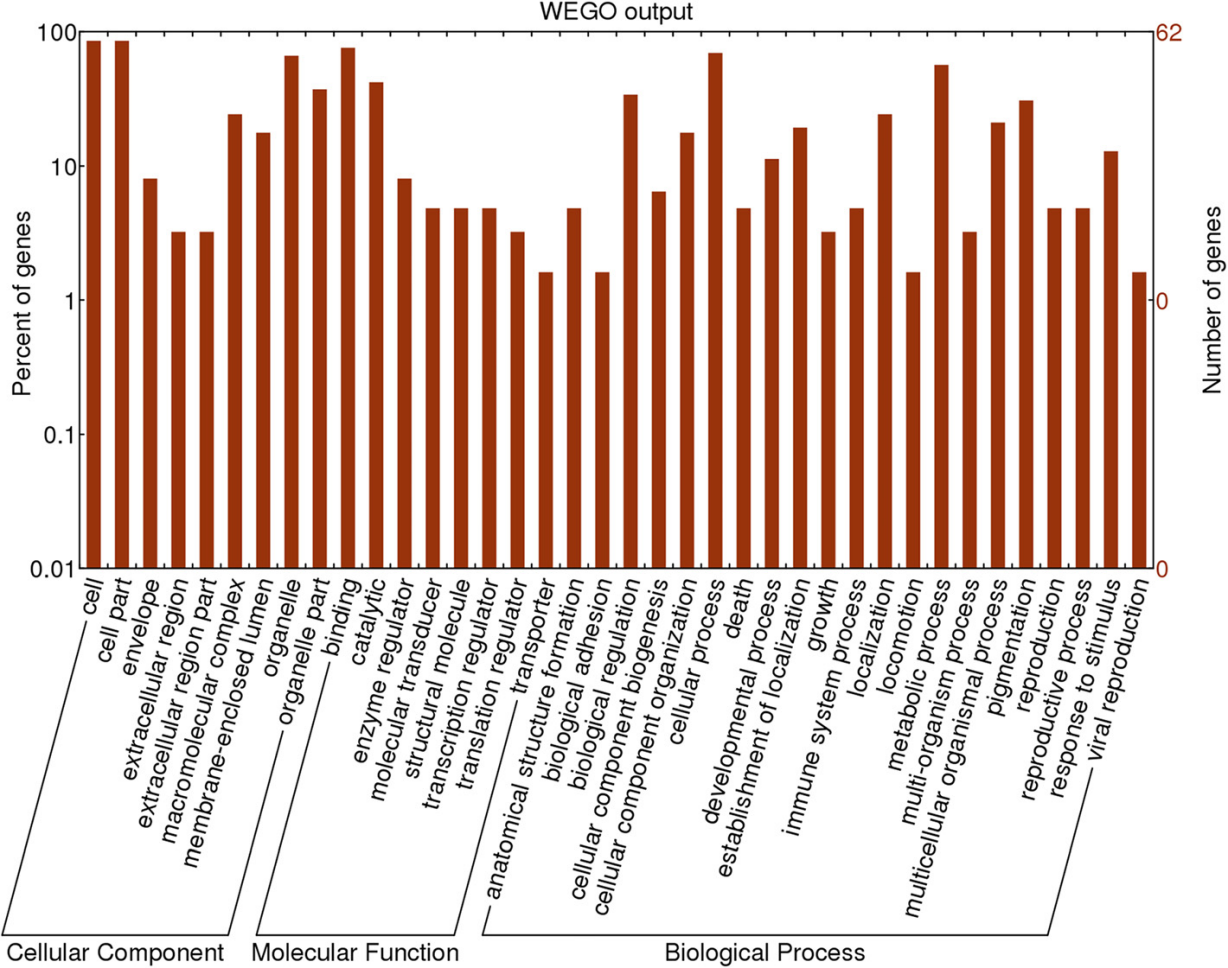
**GO distribution analysis of differentially expressed proteins in small intestinal mucosal samples from treatment group and control group.** The right coordinate axis indicates the number of proteins for each GO annotation, and the left one represents the proportion of proteins for every GO annotation.

In the cellular component group, the differentially expressed proteins are concentrated in intracellular organelles (mitochondrion, cytoskeletal part and nuclear part) and the cytoplasm part (Figure 3). In the molecular functional group, the differentially expressed proteins that are metabolic enzymes (oxidoreductase activity and hydrolase activity), binding proteins (protein binding and nucleotide binding) and enzyme regulator were ranked at the top of the category occupancy, suggesting that the relevant functions were important in the small intestinal mucosa of broilers (Figure 3). In the biological process category, the proteins that participate in cellular processes, metabolism and biological regulation were at the top ratio in the differentially expressed proteins (Figure 3), suggesting that exposure to ammonia changes the cellular metabolic process, like cellular biosynthetic process, nucleotide and nucleic acid metabolic process, alters metabolism in the intestine, such as nutrient (carbohydrate, amino acid and lipid) metabolism, and have various effects on biological processes, for example transcriptional and translational regulation, cell growth and proliferation, oxidative stress and so on.

### Validation of proteins of differential abundance

**Figure 4:**
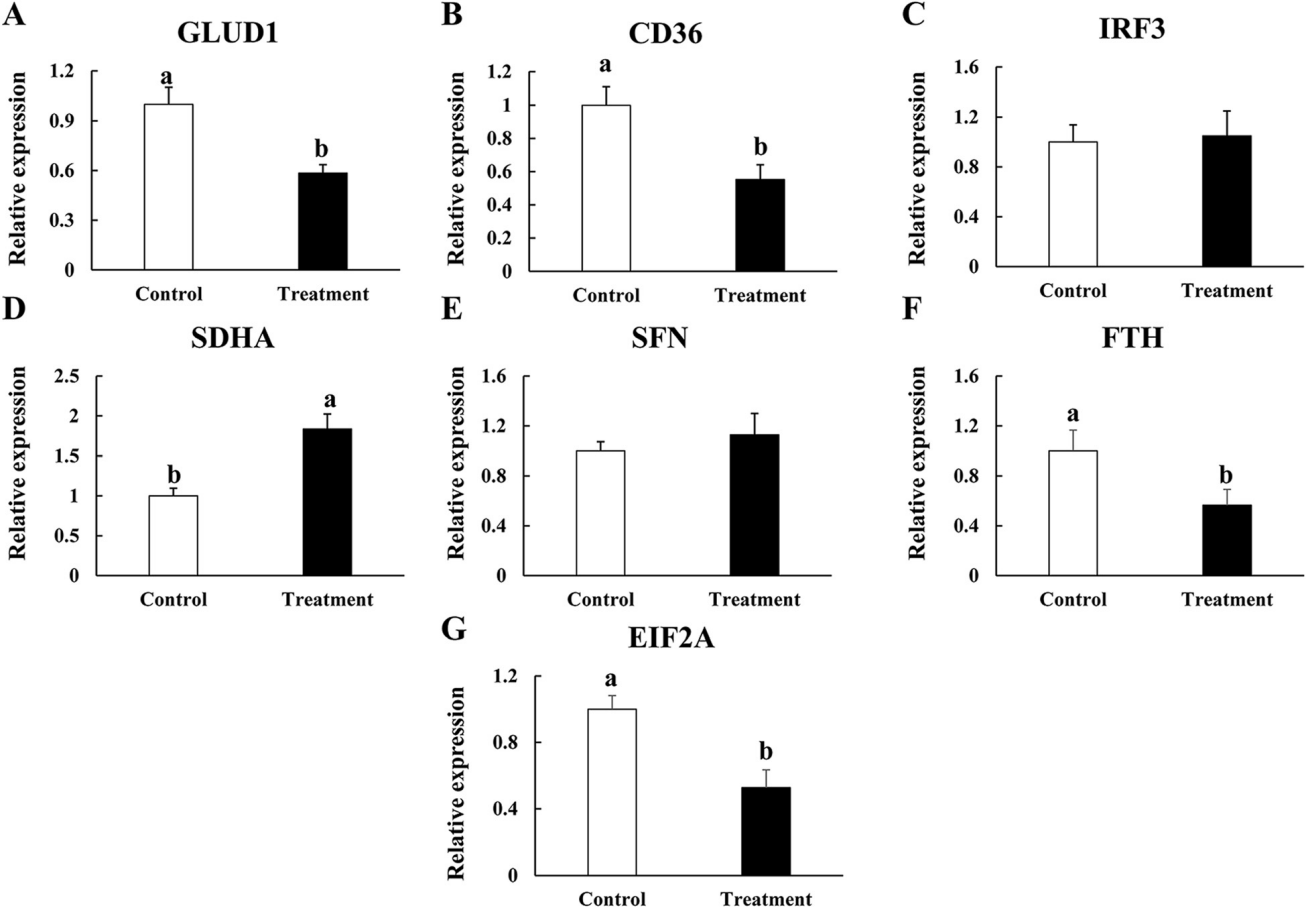
**qPCR validation of seven proteins of differential abundance from the intestinal mucosa of 42-day-old AA broilers at the mRNA level (A, B, C, D, E, F and G).** Samples were normalized with the reference gene β-actin. Vertical lines represent ± S.D, and different letters denote significant difference at *P* < 0.05 (n = 6).

Seven differentially expressed proteins (GLUD1 involved in amino acid metabolism; fatty acid translocase (CD36) involved in lipid metabolism; IRF3 involved in immune response; FTH involved in oxidative stress; SDHA involved in energy metabolism; SFN involved in cell growth and proliferation; and EIF2A involved in transcriptional and translational regulation) were selected for functional validation at the mRNA level using qPCR (Figure 4). The protein levels of GLUD1, CD36, SDHA and EIF2A were consistent with their mRNA expression levels. The results for the remaining three proteins were inconsistent between the mRNA levels and the protein levels. Possible reasons for these inconsistent results include the following: 1) the relationships between the mRNA levels and the protein levels were indirect, 2) there were some post-translational effects and/or the function of other regulatory mechanisms, and 3) there was a time delay between responses on the mRNA and protein levels [24].

## Discussion

Ammonia influences different organs and physiological functions in animals due to oxidative stress and inflammation; therefore, excess concentration of ammonia lead to plenty of health problems in the body. More and more evidence demonstrates that high concentrations of ammonia impairs energy metabolism, and induces cell apoptosis and mitochondrial damage in the mucosa of GI tract [10,12,13]. To identify the molecular mechanisms related to the exposure to high concentration of atmospheric ammonia in broilers, we compared the growth parameters, immune organ development, gut morphology, serum parameters and small intestinal mucosa proteome of control (ammonia concentration, 3 ± 3 μL/L) with those exposed to high level of ambient ammonia (ammonia concentration, 75 ± 3 μL/L). On the whole, exposure to high concentrations of ambient ammonia (75 ± 3 μL/L) greatly reduced the growth of broilers.

In addition to the reduction of growth performance, exposure to high concentrations of ammonia also resulted in interference with multiple physiological functions in broilers. As two of the most important immune organs, indices of intestine and spleen were reduced in TRET group compared to CTRL group. Moreover, ammonia-exposed chickens had much lower villus height and crypt depth among different segments of small intestine. These results indicated that exposure to high concentrations of atmospheric ammonia mainly exerts negative impacts on intestine mucosal structure and immune organ development of chickens [25], which may cause huge damages to nutrients absorption and immune system. Increased activity of serum CK and decreased activity of serum T-SOD indicated oxidative stress in ammonia-exposed broilers. Previous study also reports that obvious pathomorphological changes were observed in kidneys and livers in broiler chickens under the dynamic range of atmospheric ammonia (31–95 ppm) [8].

A total of 43 proteins related to nutrient metabolism, apoptosis, immune and oxidative response, transcriptional and translational regulation, and cell cytoskeleton and growth altered in abundances corresponding to the change in intestinal histomorphology of ammonia-exposed broilers. Of these, up-regulation proteins involved in energy metabolism and apoptosis may induce the mitochondrial apoptosis resulting in an increase rate of oxidative phosphorylation under stress, whereas down-regulation of immune and nutrient metabolic proteins may decrease the anti-microbial ability and nutrient absorption in the intestine itself.

Cytoskeletal proteins have crucial roles in the maturation, migration, and renewal of epithelial cells along the crypt-villus axis [24,34,35]. In this study, two differential protein species related to cytoskeleton were down-regulated in the small intestinal mucosa of ammonia exposed broilers. FLNA is an actin cross-linking protein that is crucial for actin cytoskeleton organization participating in cellular architectural and signaling functions [36-38]. LASP1 is a cytoskeletal adaptor protein, which has been reported as a signal molecule playing role in the differentiation of parietal cells [39,40]. This is consistent with the finding in this study that lower villus height and crypt depth among different segments of small intestine was observed in TRET group. As a result, the surface area of the intestine was decreased, and finally resulted in impairment of digestion and absorption efficiency in the gut. Other proteins involved in cell growth and proliferation, including SFN, RPL23, ZNF598 and SEPT12, are also down-regulated, and may harm the mucosal regeneration in GI tract due to ammonia exposure related injury [41-43]. Furthermore, the reduced abundance of proteins relevant to transcriptional and translational regulation, including HMGN1, DYNC2H1, EIF2S2, DENR, MMS19, RPS10, CHTOP and ARHGDIB, is observed in TRET group, which indicates a decreased capacity for protein synthesis to impair overall gut function and integrity [44-50].

Excess concentration of ammonia induces oxidative stress in various tissues, which can trigger inflammation and subsequent apoptosis [13]. In the present study, three and three differential protein species were identified in the categories of oxidative stress and apoptosis, respectively. Of these proteins, PNKD protein plays an important role in maintaining cellular redox status [51]; GLUL activity is an indicator of free radical-mediated oxidative damage in tissue injury [52]; FTH is a core subunit of iron-binding protein ferritin, which is induced to protect against oxidative stress [53,54]; ILF3 participates in apoptosis, its expression is up-regulated during apoptosis induced by H_2_O_2_ in murine macrophages [55]; COX7C is shown to represent the rate-limiting step of mitochondrial electron transport chain in normal condition [56], however, its expression cannot be controlled under oxidative stress induced apoptosis, increasing thermogenesis and the rate of oxidative phosphorylation [57]; TIMM8A is a mitochondrial intermembrane space (IMS) protein that is involved in caspase-independent cell death [58]. Down-regulation of PNKD and GLUL along with up-regulation of FTH, ILF3, COX7C and TIMM8A in the treatment group suggests that, the small intestinal mucosa of ammonia-exposed broilers are under oxidative stress, which triggers the elevation of apoptosis. Moreover, previous research has proven that oxidative stress is related to the impairment of energy metabolism [59]. In this study, proteins involved in oxidative phosphorylation, including NDUFB3, SDHA and ATHL1 [60,61], are up-regulated, and indicate that ATP production and oxidative phosphorylation are uncoupled due to oxidative stress induced by ammonia, which may explain why the feed-conversion efficiency is reduced in ammonia-exposed broilers.

As the biggest immune organ in the body, intestine plays very important roles in defense of invasion of harmful bacteria and xenobiotics [14]. In the present study, eight differential protein species related to immune response were down-regulated in the treatment group. HMGB1 and HMGB3 serve as immunogenic nucleic acids binding proteins that are generally involved in the nucleic acid receptor-mediated activation of innate immune responses [62,63]; MYO1G is a plasma membrane-associated class I myosin contributing to T-cell activation [64]; SERPINB5 is a tumor suppressor that plays a role in protein binding [65]; IRF3 is a transcription factor that plays distinct role in innate antiviral response [66]; GIT2 is one of regulators of G protein-coupled receptor (GPCR), and loss of GIT2 *in vivo* leads to an immunodeficient state [67]; HMHA1 is a major target of immune responses also playing a role in T-cell activation [68]; and PLD1 contributes to the essential function of macrophages for protecting against a wide variety of invading microorganisms [69]. Down-regulation of these proteins in the treatment group suggests that, the immunity of gut is under low condition in ammonia-exposed broilers, which increases possibilities of bacterial or viral infection and probably leads to lower growth rate.

Ammonia has been reported to interfere with nutrient metabolism in mammals, such as reduced fatty acid oxidation, vitamins and amino acids synthesis disorder, and inhibitory of gluconeogenesis [10,70,71]. In the intestinal mucosa of ammonia-exposed broilers, differential proteins involved in carbohydrate/amino acid/lipid/vitamin metabolism indicate that impairment of nutrient absorption and digestion is related to metabolic changes in the intestine, which affects gluconeogenesis, vitamin A synthesis and fatty acid metabolism.

## Conclusions

This study integrates traditional nutritional, morphological and state of the art proteomic approaches to identify the impact of high concentrations of atmospheric ammonia exposure on intestine of broilers. Reduced growth rate was observed in broilers exposed to high level of environmental ammonia. Possible reasons for exposure to ammonia derived influence on broilers are related to intestinal immune and histomorphology. Integrative data analysis indicates that exposure to high amount environmental ammonia resulted in significant changes in the development of immune organs and intestinal villi, and mucosal proteome of AA broilers. These changes might be resulting from oxidative stress induced by ammonia. Several proteins are identified to related to immune response, oxidative stress, apoptosis and mucosal structure, and thus play key roles in nutrient consumption and absorption. This study identifies the potential molecular mechanisms of high concentrations of atmospheric ammonia exposure to broilers and provides new knowledge that can be used for possible intervention using nutritional strategies in the future.

## Additional files

Additional file 1: Table S1. Composition of the experimental diet and calculated proximate composition of the diet. Additional file 2: Table S2. The qPCR primers used for verification of the differentially expressed genes of the AA broiler small intestinal mucosa. Additional file 3: Table S3. List of all proteins (n = 2726) identified in the study. Additional file 4: Table S4. List of all differently expressed proteins (n = 70) identified in the study.

## Abbreviations

CD36: Cluster of differentiation 36
SFN: Stratifin
RPL23: Ribosomal protein L23
ZNF598: Zinc finger protein 598
SEPT12: Septin 12
HMGN1: High mobility group nucleosome binding domain 1
DYNC2H1: Dynein, cytoplasmic 2, heavy chain 1
EIF2S2: Eukaryotic translation initiation factor 2, subunit 2 beta
MMS19: MMS19 nucleotide excision repair homolog
RPS10: Ribosomal protein S10
CHTOP: Chromatin target of PRMT1
ARHGDIB: Rho GDP dissociation inhibitor (GDI) beta
PNKD: Paroxysmal nonkinesigenic dyskinesia
COX7C: Cytochrome c oxidase subunit VIIc
TIMM8A: Translocase of inner mitochondrial membrane 8 homolog A
NDUFB3: NADH dehydrogenase (ubiquinone) 1 beta subcomplex, 3
ATHL1: Acid trehalase-like 1
SERPINB5: Serpin peptidase inhibitor, clade B (Ovalbumin), member 5
GIT2: G protein-coupled receptor kinase interacting ArfGAP 2
HMHA1: Histocompatibility (Minor) HA-1
PLD1: Phospholipase D1

## References

[1] NRC (2003). Air emissions from animal feeding operations: current knowledge, future needs.

[2] van Aardenne JA, Dentener FJ, Olivier JGJ, Goldewijk C, Lelieveld J (2001). A 1 degrees × 1 degrees resolution data set of historical. anthropogenic trace gas emissions for the period 1890–1990. Glob Biogeochem Cycles.

[3] US EPA (2004). National emission inventory: ammonia emissions from animal husbandry operations.

[4] Miles DM, Branton SL, Lott BD (2004). Atmospheric ammonia is detrimental to the performance of modern commercial broilers. Poult Sci.

[5] Shlomo Y (2004). Ammonia affects performance and thermoregulation of male broiler chickens. Anim Res.

[6] Sherlock L, McKeegan DE, Cheng Z, Wathes CM, Wathes DC (2012). Effects of contact dermatitis on hepatic gene expression in broilers. Br Poult Sci.

[7] Wei FX, Xu B, Hu XF, Li SY, Liu FZ, Sun QY (2012). The effect of ammonia and humidity in poultry houses on intestinal morphology and function of broilers. J Anim Vet Adv.

[8] Witkowska D, Sowinska J, Iwanczuk Czernik K, Mituniewicz T, Wojcik A, Szarek J (2006). The effect of a disinfectant on the ammonia concentration on the surface of litter, air and the pathomorphological picture of kidneys and livers in broiler chickens. Archiv fur Tierzucht.

[9] Bobermin LD, Quincozes-Santos A, Guerra MC, Leite MC, Souza DO, Gonçalves CA (2012). Resveratrol prevents ammonia toxicity in astroglial cells. PLoS One.

[10] Cremin JD, Fitch MD, Fleming SE (2003). Glucose alleviates ammonia-induced inhibition of short-chain fatty acid metabolism in rat colonic epithelial cells. Am J Physiol Gastrointest Liver Physiol.

[11] Andriamihaja M, Davila AM, Eklou-Lawson M, Petit N, Delpal S, Allek F (2010). Colon luminal content and epithelial cell morphology are markedly modified in rats fed with a high-protein diet. Am J Physiol Gastrointest Liver Physiol.

[12] Tsujii M, Kawano S, Tsuji S, Fusamoto H, Kamada T, Sato N (1992). Mechanism of gastric mucosal damage induced by ammonia. Gastroenterology.

[13] Igarashi M, Kitada Y, Yoshiyama H, Takagi A, Miwa T, Koga Y (2001). Ammonia as an accelerator of tumor necrosis factor alpha-induced apoptosis of gastric epithelial cells in Helicobacter pylori infection. Infect Immun.

[14] Furness JB, Kunze WA, Clerc N (1999). Nutrient tasting and signaling mechanisms in the gut. II. The intestine as a sensory organ: neural, endocrine, and immune responses. Am J Physiol.

[15] Wang X, Yang F, Liu C, Zhou H, Wu G, Qiao S (2012). Dietary supplementation with the probiotic Lactobacillus fermentum I5007 and the antibiotic aureomycin differentially affects the small intestinal proteomes of weanling piglets. J Nutr.

[16] Wang X, Ou D, Yin J, Wu G, Wang J (2009). Proteomic analysis reveals altered expression of proteins related to glutathione metabolism and apoptosis in the small intestine of zinc oxide-supplemented piglets. Amino Acids.

[17] Soler L, Niewold TA, Moreno Á, Garrido JJ (2014). Proteomic approaches to study the pig intestinal system. Curr Protein Pept Sci.

[18] Choi PM, Guo J, Erwin CR, Wandu WS, Leinicke JA, Xie Y (2014). Disruption of retinoblastoma protein expression in the intestinal epithelium impairs lipid absorption. Am J Physiol Gastrointest Liver Physiol.

[19] Ahmad MK, Khan AA, Mahmood R (2012). Alterations in brush border membrane enzymes, carbohydrate metabolism and oxidative damage to rat intestine by potassium bromate. Biochimie.

[20] Keszthelyi D, Troost FJ, Jonkers DM, van Eijk HM, Lindsey PJ, Dekker J (2014). Serotonergic reinforcement of intestinal barrier function is impaired in irritable bowel syndrome. Aliment Pharmacol Ther.

[21] Li C, Li Q, Liu YY, Wang MX, Pan CS, Yan L (2014). Protective effects of Notoginsenoside R1 on intestinal ischemia-reperfusion injury in rats. Am J Physiol Gastrointest Liver Physiol.

[22] Kleinman MT, Araujo JA, Nel A, Sioutas C, Campbell A, Cong PQ (2008). Inhaled ultrafine particulate matter affects CNS inflammatory processes and may act via MAP kinase signaling pathways. Toxicol Lett.

[23] Huang J, Zhang Y, Zhou Y, Zhang Z, Xie Z, Zhang J (2013). Green tea polyphenols alleviate obesity in broiler chickens through the regulation of lipid-metabolism-related genes and transcription factor expression. J Agric Food Chem.

[24] Luo J, Zheng A, Meng K, Chang W, Bai Y, Li K (2013). Proteome changes in the intestinal mucosa of broiler (Gallus gallus) activated by probiotic Enterococcus faecium. J Proteomics.

[25] Uni Z, Gal-Garber O, Geyra A, Sklan D, Yahav S (2001). Changes in growth and function of chick small intestine epithelium due to early thermal conditioning. Poult Sci.

[26] Diz AP, Truebano M, Skibinski DO (2009). The consequences of sample pooling in proteomics: an empirical study. Electrophoresis.

[27] Su L, Cao L, Zhou R, Jiang Z, Xiao K, Kong W (2013). Identification of novel biomarkers for sepsis prognosis via urinary proteomic analysis using iTRAQ labeling and 2D-LC-MS/MS. PLoS One.

[28] Olsen JV, Blagoev B, Gnad F, Macek B, Kumar C, Mortensen P (2006). Global, in vivo, and site-specific phosphorylation dynamics in signaling networks. Cell.

[29] Hakimov HA, Walters S, Wright TC, Meidinger RG, Verschoor CP, Gadish M (2009). Application of iTRAQ to catalogue the skeletal muscle proteome in pigs and assessment of effects of gender and diet dephytinization. Proteomics.

[30] Ye J, Fang L, Zheng H, Zhang Y, Chen J, Zhang Z (2006). WEGO: a web tool for plotting GO annotations. Nucleic Acids Res.

[31] Zi J, Zhang J, Wang Q, Zhou B, Zhong J, Zhang C (2013). Stress responsive proteins are actively regulated during rice (Oryza sativa) embryogenesis as indicated by quantitative proteomics analysis. PLoS One.

[32] Chulayo AY, Muchenje V (2013). The effects of pre-slaughter stress and season on the activity of plasma creatine kinase and mutton quality from different sheep breeds slaughtered at a smallholder abattoir. Asian-Australas J Anim Sci.

[33] Dong XY, Azzam MM, Rao W, Yu DY, Zou XT (2012). Evaluating the impact of excess dietary tryptophan on laying performance and immune function of laying hens reared under hot and humid summer conditions. Br Poult Sci.

[34] Di Garbo A, Johnston MD, Chapman SJ, Maini PK (2010). Variable renewal rate and growth properties of cell populations in colon crypts. Phys Rev E Stat Nonlin Soft Matter Phys.

[35] Gordon JI, Hermiston ML (1994). Differentiation and self-renewal in the mouse gastrointestinal epithelium. Curr Opin Cell Biol.

[36] van der Flier A, Sonnenberg A (2001). Structural and functional aspects of filamins. Biochim Biophys Acta.

[37] Tu Y, Wu S, Shi X, Chen K, Wu C (2003). Migfilin and Mig-2 link focal adhesions to filamin and the actin cytoskeleton and function in cell shape modulation. Cell.

[38] Stossel TP, Condeelis J, Cooley L, Hartwig JH, Noegel A, Schleicher M (2001). Filamins as integrators of cell mechanics and signalling. Nat Rev Mol Cell Biol.

[39] Iiizumi G, Sadoya Y, Hino S, Shibuya N, Kawabata H (2007). Proteomic characterization of the site-dependent functional difference in the rat small intestine. Biochim Biophys Acta.

[40] Jain RN, Samuelson LC (2006). Differentiation of the gastric mucosa. II. Role of gastrin in gastric epithelial cell proliferation and maturation. Am J Physiol Gastrointest Liver Physiol.

[41] Murphy EF, Hooiveld GJ, Muller M, Calogero RA, Cashman KD (2007). Conjugated linoleic acid alters global gene expression in human intestinal-like Caco-2 cells in an isomer-specific manner. J Nutr.

[42] Wanzel M, Russ AC, Kleine-Kohlbrecher D, Colombo E, Pelicci PG, Eilers M (2008). A ribosomal protein L23-nucleophosmin circuit coordinates Mizl function with cell growth. Nat Cell Biol.

[43] Morita M, Ler LW, Fabian MR, Siddiqui N, Mullin M, Henderson VC (2012). A novel 4EHP-GIGYF2 translational repressor complex is essential for mammalian development. Mol Cell Biol.

[44] Ostergaard M, Hansen GA, Vorum H, Honoré B (2006). Proteomic profiling of fibroblasts reveals a modulating effect of extracellular calumenin on the organization of the actin cytoskeleton. Proteomics.

[45] Birger Y, West KL, Postnikov YV, Lim JH, Furusawa T, Wagner JP (2003). Chromosomal protein HMGN1 enhances the rate of DNA repair in chromatin. EMBO J.

[46] Schmidts M, Arts HH, Bongers EM, Yap Z, Oud MM, Antony D (2013). Exome sequencing identifies DYNC2H1 mutations as a common cause of asphyxiating thoracic dystrophy (Jeune syndrome) without major polydactyly, renal or retinal involvement. J Med Genet.

[47] Skabkin MA, Skabkina OV, Dhote V, Komar AA, Hellen CU, Pestova TV (2010). Activities of Ligatin and MCT-1/DENR in eukaryotic translation initiation and ribosomal recycling. Genes Dev.

[48] van Wietmarschen N, Moradian A, Morin GB, Lansdorp PM, Uringa EJ (2012). The mammalian proteins MMS19, MIP18, and ANT2 are involved in cytoplasmic iron-sulfur cluster protein assembly. J Biol Chem.

[49] Fanis P, Gillemans N, Aghajanirefah A, Pourfarzad F, Demmers J, Esteghamat F (2012). Five friends of methylated chromatin target of protein-arginine-methyltransferase[prmt]-1 (chtop), a complex linking arginine methylation to desumoylation. Mol Cell Proteomics.

[50] Marc Rhoads J, Wu G (2009). Glutamine, arginine, and leucine signaling in the intestine. Amino Acids.

[51] Shen Y, Lee HY, Rawson J, Ojha S, Babbitt P, Fu YH (2011). Mutations in PNKD causing paroxysmal dyskinesia alters protein cleavage and stability. Hum Mol Genet.

[52] Oliver CN, Starke-Reed PE, Stadtman ER, Liu GJ, Carney JM, Floyd RA (1990). Oxidative damage to brain proteins, loss of glutamine synthetase activity, and production of free radicals during ischemia/reperfusion-induced injury to gerbil brain. Proc Natl Acad Sci U S A.

[53] Aung W, Hasegawa S, Furukawa T, Saga T (2007). Potential role of ferritin heavy chain in oxidative stress and apoptosis in human mesothelial and mesothelioma cells: implications for asbestos-induced oncogenesis. Carcinogenesis.

[54] Polyzos S, Kountouras J, Zavos C, Papatheodorou A, Katsiki E, Patsiaoura K (2012). Serum ferritin in patients with nonalcoholic fatty liver disease: evaluation of ferritin to adiponectin ratio and ferritin by homeostatic model of assessment insulin resistance product as non-invasive markers. Immuno-Gastroenterology.

[55] Fong CC, Zhang Y, Zhang Q, Tzang CH, Fong WF, Wu RS (2007). Dexamethasone protects RAW264.7 macrophages from growth arrest and apoptosis induced by H_2_O_2_ through alteration of gene expression patterns and inhibition of nuclear factor-kappa B (NF-kappaB) activity. Toxicology.

[56] Villani G, Greco M, Papa S, Attardi G (1998). Low reserve of cytochrome c oxidase capacity in vivo in the respiratory chain of a variety of human cell types. J Biol Chem.

[57] Kadenbach B, Arnold S, Lee I, Hüttemann M (2004). The possible role of cytochrome c oxidase in stress-induced apoptosis and degenerative diseases. Biochim Biophys Acta.

[58] Arnoult D, Rismanchi N, Grodet A, Roberts RG, Seeburg DP, Estaquier J (2005). Bax/Bak-dependent release of DDP/TIMM8a promotes Drp1-mediated mitochondrial fission and mitoptosis during programmed cell death. Curr Biol.

[59] Zaza G, Granata S, Masola V, Rugiu C, Fantin F, Gesualdo L (2013). Downregulation of nuclear-encoded genes of oxidative metabolism in dialyzed chronic kidney disease patients. PLoS One.

[60] Sparks LM, Xie H, Koza RA, Mynatt R, Hulver MW, Bray GA (2005). A high-fat diet coordinately downregulates genes required for mitochondrial oxidative phosphorylation in skeletal muscle. Diabetes.

[61] Sokolović M, Wehkamp D, Sokolović A, Vermeulen J, Gilhuijs-Pederson LA, van Haaften RI (2007). Fasting induces a biphasic adaptive metabolic response in murine small intestine. BMC Genomics.

[62] Yanai H, Ban T, Taniguchi T (2011). Essential role of high-mobility group box proteins in nucleic acid-mediated innate immune responses. J Intern Med.

[63] Zhang Q, Wang Y (2008). High mobility group proteins and their post-translational modifications. Biochim Biophys Acta.

[64] Lopez GP, Ostap EM, Shaw S (2009). Myosin 1G is a hematopoietic-restricted protein highly enriched in lymphocyte plasma membrane/microvilli whose deficiency impairs lymphocyte activation. J Immunol.

[65] Ding Y, Lu B, Chen D, Meng L, Shen Y, Chen S (2010). Proteomic analysis of colonic mucosa in a rat model of irritable bowel syndrome. Proteomics.

[66] Collins SE, Noyce RS, Mossman KL (2004). Innate cellular response to virus particle entry requires IRF3 but not virus replication. J Virol.

[67] Mazaki Y, Hashimoto S, Tsujimura T, Morishige M, Hashimoto A, Aritake K (2006). Neutrophil direction sensing and superoxide production linked by the GTPase-activating protein GIT2. Nat Immunol.

[68] Nicholls S, Piper KP, Mohammed F, Dafforn TR, Tenzer S, Salim M (2009). Secondary anchor polymorphism in the HA-1 minor histocompatibility antigen critically affects MHC stability and TCR recognition. Proc Natl Acad Sci U S A.

[69] Tian Y, Pate C, Andreolotti A, Wang L, Tuomanen E, Boyd K (2008). Cytokine secretion requires phosphatidylcholine synthesis. J Cell Biol.

[70] Comar JF, Suzuki-Kemmelmeier F, Constantin J, Bracht A (2010). Hepatic zonation of carbon and nitrogen fluxes derived from glutamine and ammonia transformations. J Biomed Sci.

[71] Essa MM, Subramanian P (2006). Pongamia pinnata modulates the oxidant-antioxidant imbalance in ammonium chloride-induced hyperammonemic rats. Fundam Clin Pharmacol.

