# Supplementary material for: Proteome changes in the small intestinal mucosa of broilers (Gallus gallus) induced by high concentrations of atmospheric ammonia
Source: Proteome Sci. 2015 Feb 21;13:9. doi: 10.1186/s12953-015-0067-4 (PMC4347970; doi:10.1186/s12953-015-0067-4)
Supplement: Additional file 2: Table S2. — The qPCR primers used for verification of the differentially expressed genes of the AA broiler small intestinal mucosa. [file 12953_2015_67_MOESM2_ESM.docx]

**Table S2 The qPCR primers used for verification of the differentially expressed genes of the AA broiler hepatic tissues**

| Gene name | Primer sequence | Product length（bp） |
| --- | --- | --- |
| β-actin | forward: GAGAAATTGTGCGTGACATCA  reverse: CCTGAACCTCTCATTGCCA | 152 |
| EIF2A | forward: GACGTGGACAAAACAGGTGC  reverse: AGGTGGAATCGGAGGCTACT | 361 |
| SFN | forward: TCATCTCCAGCATCGAGCAC  reverse: CTCTTGGTACGCCTTCTGGG | 272 |
| SDHA | forward: CTTGGCTAAGTGTTGGCTGC  reverse: AAGCTGCATTGATCCCTCCC | 305 |
| FHC | forward: GCTGCACAAATTGGCAACTG  reverse: CCATCTTCCGCAGGTTGGTC | 128 |
| IRF3 | forward: CTCAACCACCACGATTTGGC  reverse: GGGTTGGCAGACTGGAAGAG | 392 |
| CD36 | forward:: GTCTGCACCCTGTCAAAGGA  reverse: AGGCACGTGATGGTGTCATT | 435 |
| GLUD1 | forward: GTAAACCCATCAGCCAGGGT  reverse: CAAGCTTCTGCGCTTTAGGG | 333 |

EIF2A = eukaryotic translation initiation factor 2A; SFN = stratifin; SDHA = succinate dehydrogenase [ubiquinone] flavoprotein subunit, mitochondrial; FTH = ferritin heavy chain; IRF3 = interferon regulatory factor 3; CD36 = cluster of differentiation 36; GLUD1 = glutamate dehydrogenase 1
